# *RTEL1* polymorphisms are associated with lung cancer risk in the Chinese Han population

**DOI:** 10.18632/oncotarget.12297

**Published:** 2016-09-28

**Authors:** Shouchun Yan, Ridong Xia, Tianbo Jin, Hui Ren, Hua Yang, Jing Li, Mengdan Yan, Yuanyuan Zhu, Mingwei Chen

**Affiliations:** ^1^ Department of Respiratory and Critical Care Medicine, The First Affiliated Hospital of Xi'an Jiaotong University, Xi'an, Shaanxi 710061, China; ^2^ Department of Emergency Medicine, Xi'an NO.1 Hospital, Xi'an, Shaanxi 710002, China; ^3^ Key Laboratory of Molecular Mechanism and Intervention Research for Plateau Diseases of Tibet Autonomous Region, School of Medicine, Xizang Minzu University, Xianyang, Shaanxi 712082, China; ^4^ Key Laboratory of High Altitude Environment and Genes Related to Diseases of Tibet Autonomous Region, School of Medicine, Xizang Minzu University, Xianyang, Shaanxi 712082, China; ^5^ Key Laboratory for Basic Life Science Research of Tibet Autonomous Region, School of Medicine, Xizang Minzu University, Xianyang, Shaanxi 712082, China; ^6^ School of Life Sciences, Northwest University, Xi'an, Shaanxi 710069, China

**Keywords:** single nucleotide polymorphism, RTEL1, lung cancer, case-control study, Chinese Han

## Abstract

*RTEL1* (regulator of telomere elongation helicase 1; OMIM 608833) gene polymorphisms were linked to lung cancer (LC) susceptibility in a cancer genome-wide association study (GWAS) Here, we assessed whether seven previously reported *RTEL1* polymorphisms influenced LC risk in Han Chinese population. All study samples (554 LC cases and 696 cancer-free controls) were collected from the Affiliated Hospital of Xizang Minzu University in China. We assessed associations between SNPs and LC risk using various several genetic models (codominant, dominant, recessive, overdominant, and additive). Whereas rs2738780 showed a protective effect against LC (Odds ratio (OR) = 0.80 ;95% confidence interval (CI): 0.638 = 0.998; *p* = 0.048), rs7261546(OR = 4.16; 95% CI: 1.35–12.82; *p* = 0.007), rs6062299(OR=5.08; 95% CI: 1.43–18.10; *p* = 0.005) and rs3787098(OR = 5.10; 95% CI: 1.43–18.15; *p* = 0.004) were all associated with increased LC susceptibility (recessive model). Haplotype analysis suggested that “CTC” was associated with a 0.8-fold decrease in LC risk (OR = 0.80, 95% CI, 0.63–1.00; Pearson's *p* = 0.05). These findings suggest a potential association between *RTEL1* polymorphisms and LC risk in a Chinese Han population.

## INTRODUCTION

Lung Cancer (LC) is differentiated into four major histologic classes, including adenocarcinoma, squamous cell carcinoma, small cell carcinoma and large cell carcinoma [1]. LC is the leading cause of cancer mortality worldwide, with a death rate in China of 39.54/100,000 [2]. Despite recent chemotherapy advances, there is no effective method for treating LC [3]

Causes of lung cancer are diverse, and include active and passive smoking, ionizing radiation exposure, arsenic, aromatic hydrocarbons, genetic predisposition, lifestyle, diet and pre-existing nonmalignant lung diseases, such as idiopathic pulmonary fibrosis and tuberculosis [4]. Genetic predisposition frequently plays an extremely important role in the occurrence of cancer. Incidences of rare alleles are significantly higher in cancer patients than controls, suggesting that these alleles predisposed patients to tumors [5, 6]. Multiple *RTEL1* (regulator of telomere elongation helicase 1) gene mutants appear e relevant to LC [7].

*RTEL1* plays a crucial role in cancers, including LC, and in hereditary diseases, such as Hoyeraal–Hreidarsson syndrome [8, 9]. Cancer genome-wide association studies (GWAS) showed that *RTEL1* polymorphisms contribute to LC risk [10–12]. *RTEL1* SNPs, rs2297434, rs7261546, rs2738780, rs6062299, s2777937, rs3787098 and rs2297440, have not yet been studied with respect to LC. The present study was performed to evaluate the association of these seven SNPs with LC risk in the Chinese population.

## RESULTS

### Subject characteristics

The study was carried out on 554 LC patients (138 female, 24.9%; 416 male, 75.1%) and 696 controls (304 female, 43.7%; 392 male, 56.3%). Median age was 58.1 years (24–85) for the case group and 48.6 years (18–82) for the control group. For smoking status, individuals who smoked <2 cigarettes per week or <100 per year were defined as non-smoking; otherwise, individuals were considered smoking. For alcohol drinking, more than once per week in the past six months was considered to be drinking; otherwise, individuals were considered non-drinking.We found that gender, age and smoking status, but not drinking status, was significant in both groups (*p* < 0.05, Table 1).

**Table 1:** Distributions of select characteristics by case-control status

Variable	Cases (n = 554)	Controls (n = 696)	*p*-value
Sex			*p* < 0.001^a^
Male	416	392	
Female	138	304	
Smoking status			*p* < 0.001^a^
Smoking	352	293	
Non-Smoking	202	403	
Drinking status			0.35
Drinking	147	173	
Non-drinking	407	423	
Age, year (mean ± SD)	58.1 ± 10.5	48.6 ± 9.5	*p* < 0.05^b^

a *p* values were calculated from Chi-square tests.

b *p* values were calculated by Student' *t* tests.

### Associations between individual SNPs and LC risk

We tested six SNPs for association with LC risk in cases and controls. One SNP (rs2297440) was excluded for deviation from Hardy-Weinberg equilibrium (*p* < 0.01). We used the chi-squared test to assess the influence of gene polymorphism on LC risk in the allele model, and found that *RTEL1* rs2738780 reduced LC risk OR = 0.80 95% CI: 0.638–0.998, *p* = 0.048, Table 2).

**Table 2:** *RTEL1* SNPs analyzed in this study

SNP ID	Gene	Chromosome position	Base change	MAF—case	MAF—control	HWE *p*-value	OR (95% CI)	*p* value
rs2297434	RTEL1	20.00	T/C	0.13	0.12	1.000	1.051 (0.826–1.335)	0.688
rs7261546	RTEL1	20.00	G/C	0.13	0.12	0.043	1.145 (0.903–1.453)	0.264
rs2738780	RTEL1	20.00	T/C	0.14	0.16	0.405	0.798 (0.638–0.998)	0.048*
rs6062299	RTEL1	20.00	C/G	0.13	0.12	0.015	1.135 (0.892–1.443)	0.302
rs2777937	RTEL1	20.00	T/C	0.13	0.12	1.000	1.045 (0.822–1.327)	0.719
rs3787098	RTEL1	20.00	A/G	0.12	0.11	0.068	1.141 (0.888–1.465)	0.303
rs2297440	RTEL1	20.00	C/T	0.26	0.24	0.0122	1.149 (0.958–1.379)	0.135

* Site with HWE *P* ≤ 0.01 excluded. HWE, Hardy–Weinberg equilibrium; MAF, minor allele frequency; OR, odds ratio; CI, confidence interval; SNP, single-nucleotide polymorphism. *p ≤ 0.05 indicates statistical significance.

We also used unconditional logistic regression analysis in five genetic models (co-dominant, dominant, recessive, over-dominant and log-additive) to appraise the association between each SNP and LC risk (Table 3). The best inheritance model was assessed using Akaike information criteria (AIC) and Bayesian information criteria (BIC), with the model with the lowest values being the best fit [13]. ‘C/C-G/C’ of rs7261546, ‘C/C’ of rs6062299 and ‘A/A’ of rs3787098 increased LC risk, and exerted a recessive effect (*p* = 0.007, OR = 4.16, 95% CI: 1.35–12.82; *p* = 0.005, OR = 5.08, 95% CI: 1.43–18.10; *p* = 0.004, OR = 5.10, 95% CI: 1.43–18.15, respectively; Table 3). In the additive model, allele ‘G’ in rs2853672 had a protective effect (*p* = 0.044, OR = 0.66, 95% CI: 0.45–0.97).

**Table 3:** Relationship between rs2297440 alleles and LC risk in different genetic models

SNP	Model	Genotype	group = control	group = case	OR (95% CI)	*p*-value
rs7261546	Codominant	C/C	536 (77%)	420 (75.8%)	1	0.025
		G/C	156 (22.4%)	121 (21.8%)	0.99 (0.76–1.30)	
		G/G	4 (0.6%)	13 (2.4%)	4.15 (1.34–12.81)	
	Dominant	C/C	536 (77%)	420 (75.8%)	1	0.62
		G/C-G/G	160 (23%)	134 (24.2%)	1.07 (0.82–1.39)	
	Recessive	C/C-G/C	692 (99.4%)	541 (97.7%)	1	0.007*
		G/G	4 (0.6%)	13 (2.4%)	4.16 (1.35–12.82)	
	Overdominant	C/C-G/G	540 (77.6%)	433 (78.2%)	1	0.81
		G/C	156 (22.4%)	121 (21.8%)	0.97 (0.74–1.27)	
	Log-additive	---	---	---	1.15 (0.90–1.46)	0.26
rs2738780	Codominant	C/C	481 (69.4%)	410 (74.4%)	1	0.13
		T/C	197 (28.4%)	133 (24.1%)	0.79 (0.61–1.02)	
		T/T	15 (2.2%)	8 (1.4%)	0.63 (0.26–1.49)	
	Dominant	C/C	481 (69.4%)	410 (74.4%)	1	0.051
		T/C-T/T	212 (30.6%)	141 (25.6%)	0.78 (0.61–1.00)	
	Recessive	C/C-T/C	678 (97.8%)	543 (98.5%)	1	0.35
		T/T	15 (2.2%)	8 (1.4%)	0.67 (0.28–1.58)	
	Overdominant	C/C-T/T	496 (71.6%)	418 (75.9%)	1	0.088
		T/C	197 (28.4%)	133 (24.1%)	0.80 (0.62–1.03)	
	Log-additive	---	---	---	0.79 (0.63–0.99)	0.044*
rs6062299	Codominant	G/G	534 (77.2%)	422 (76.2%)	1	0.018
		G/C	155 (22.4%)	120 (21.7%)	0.98 (0.75–1.28)	
		C/C	3 (0.4%)	12 (2.2%)	5.06 (1.42–18.04)	
	Dominant	G/G	534 (77.2%)	422 (76.2%)	1	0.68
		G/C-C/C	158 (22.8%)	132 (23.8%)	1.06 (0.81–1.38)	
	Recessive	G/G-G/C	689 (99.6%)	542 (97.8%)	1	0.005*
		C/C	3 (0.4%)	12 (2.2%)	5.08 (1.43–18.10)	
	Overdominant	G/G-C/C	537 (77.6%)	434 (78.3%)	1	0.75
		G/C	155 (22.4%)	120 (21.7%)	0.96 (0.73–1.25)	
	Log-additive	---	---	---	1.14 (0.89–1.45)	0.3
rs3787098	Codominant	G/G	551 (79.4%)	435 (78.5%)	1	0.017
		G/A	140 (20.2%)	107 (19.3%)	0.97 (0.73–1.28)	
		A/A	3 (0.4%)	12 (2.2%)	5.07 (1.42–18.06)	
	Dominant	G/G	551 (79.4%)	435 (78.5%)	1	0.71
		G/A-A/A	143 (20.6%)	119 (21.5%)	1.05 (0.80–1.39)	
	Recessive	G/G-G/A	691 (99.6%)	542 (97.8%)	1	0.004*
		A/A	3 (0.4%)	12 (2.2%)	5.10 (1.43–18.15)	
	Overdominant	G/G-A/A	554 (79.8%)	447 (80.7%)	1	0.7
		G/A	140 (20.2%)	107 (19.3%)	0.95 (0.72–1.25)	
	Log-additive	---	---	---	1.14 (0.89–1.47)	0.3

OR, odd ratio; CI, confidence interval AIC, Akaike's information criterion; BIC, Bayesian information criterion

* *p* ≤ 0.05 indicates statistical significance.

### Linkage disequilibrium of *RTEL1* SNPs

**Figure 1:**
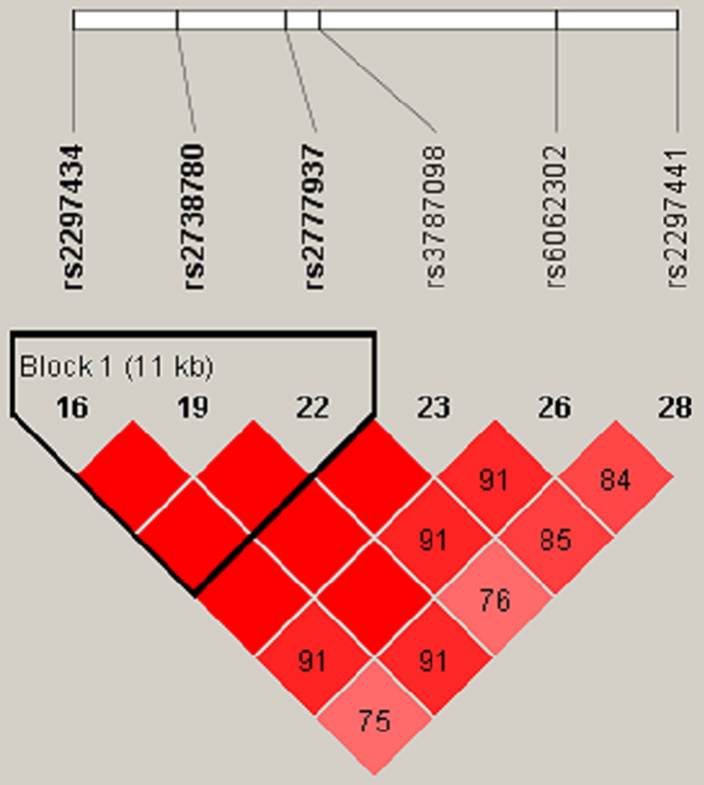
Linkage disequilibrium (LD) of all *RTEL1* polymorphic sites Brighter red represents stronger LD (LOD = 2, D′ = 1). Numbers inside boxes represent LD r^2^ values.

Linkage disequilibrium (LD) analysis identified three haplotype blocks on chromosome 20, and three *RTEL1* SNPs formed a block within 11 kb (chr: 2297434–2777937). *RTEL1* SNP LD patterns by Haploview analysis are shown in Figure 1. We investigated associations between different haplotypes using the three *RTEL1* SNPs (rs2297434 rs2738780 rs2777937) and found that the haplotype “CTC” was associated with 0.8-fold decreased LC risk (OR = 0.80, 95% CI, 0.63–1.00, Pearson's *p* = 0.05) Table 4.

**Table 4:** *RTEL1* haplotypes and their association with LC risk

Haplotype	Freq	OR (95% CI)	*p*-value
CCC	0.7255	1	---
CTC	0.1501	0.80 (0.63–1.00)	0.05*
TCT	0.1224	1.00 (0.78–1.26)	0.97
rare	0.002	1.78 (0.13–23.93)	0.66

Global haplotype association *p*-value: 0.23

CI, confidence interval; OR, odd ratio

* *p* ≤ 0.05 indicates statistical significance.

## DISCUSSION

RTEL1 is an ATP-dependent DNA helicase that was initially identified in mice as a dominant telomere length regulator [14]. Telomeres are guanine-rich tandem DNA repeats (TTAGGG repeats in mammals) located at the ends of linear chromosomes. The shelterin complex is essential to protect telomeres from degradation and from DNA repair mechanisms that might otherwise mistakenly remove and “repair” telomeric DNA [15]. Human telomeres are about 10–15 kb in length and are shortened by 30 to 200 bp each cell division, due to incomplete replication of linear DNA molecules and the absence of a chromosome end replication mechanism [16]. Telomere shortening may underlie chromosome instability in tumor cells. Multiple lines of evidence support the hypothesis that telomere dysfunction promotes genetic changes required for tumorigenesis and malignant progression [17]. In the absence of *R*TEL1, telomeres are not maintained and chromosome fusions are observed.

In our case-control study, we evaluated seven SNPs associated with LC susceptibility in a Chinese Han population and our found that rs2738780 and the “CTC” haplotype are associated with decreased LC risk. The *R*TEL1 gene, located on 20q13.3, encodes an essential helicase that plays a crucial role in mitotic and meiotic DNA double-stranded break repair [18, 20]. Double-stranded break functional consequences include oncogene activation, as in many leukemias, lymphomas and sarcomas, and tumor suppressor loss or inactivation, as in many solid tumors and most carcinomas [21, 22]. *RTEL1* is expected to act as a tumor suppressor. However, as indicated by human genetic data, the *RTEL1* genomic locus (20q13.3) is frequently amplified in human cancers [23–25], and ted *RTEL1* upregulation could be important for tumorigenesis. Cancer GWAS have linked *RTEL1* mutation to LC [10–12].

*R*TEL1 rs3787098 allele frequency has been associated with glioblastoma risk in the Chinese Han population [26]. In the current study, we found that genotype ‘A/A’ of rs3787098 was associated with increased LC risk. How SNP rs3787098 affects pathogenesis in various cancer types still needs further study. In a meta-analysis, Zhang, *et al.* [27] found that the rs2297440 polymorphism moderately increased glioma risk in all genetic models.

Some limitations in our case–control study were notable. First, the sample size was relatively small. Second, correlations between genetic polymorphisms and clinicopathologic types (squamous cell carcinoma, adenocarcinoma and other histological LC types) were not considered. These limitations will be addressed in future studies.

In conclusion, our study provides new evidence of the relationship between *RTEL1* and LC, and specific *RTEL1* SNPs may increase or decrease LC risk in the Chinese Han population.

## MATERIALS AND METHODS

### Study population and procedures

Research subjects included 554 lung cancer patients and 696 cancer-free controls. Patients were recruited from January 2011 to February 2015 at the Affiliated Hospital of the Xizang Minzu University in China. Lung cancer patients had no history of other malignancy or prior chemotherapy or radiotherapy. Controls had no acute or chronic pathology. Their cancer-free state was confirmed by testing carcinoembryonic antigen and α-fetoprotein plasma levels. The study protocol was approved by the Institutional Research Ethic Committee of the Affiliated Hospital of the Xizang Minzu University in China, Xizang Minzu University and written consent was obtained from all subjects. Two ml of whole blood was collected from each patient in K2 EDTA tubes and stored at −20°C for further study.

### SNP selection and genotyping

Based on SNPs with minor allele frequencies (MAF) greater than 0.05 identified in a prior LC association analysis of a CHB (Han Chinese in Beijing, China) population, we chose seven *RTEL1* SNPs for further genotyping. We used the genomic DNA purification kit (GoldMag Co. Ltd., Xi'an, China) to extract DNA from peripheral blood samples. DNA sample concentrations and purities were assessed by absorbance at 260 nm with a NanoDrop 2000 (Thermo Scientific, Waltham, MA). Contamination by proteins was assessed by measuring absorbance at 280 nm and calculating 260/280 ratios. MassARRAY Assay Design 3.0 Software (Sequenom, San Diego, CA, USA) was used to design the PCR assay and iPLEX single-base extension primers for the Multiplexed SNP MassEXTEND assay [28]. Sequenom Typer 4.0 software (Sequenom) was used for data management and analyses [29].

### Statistical analysis

The statistical analysis was performed using SPSS 17.0 software (SPSS, Inc.). Hardy–Weinberg equilibrium (HWE) was tested separately for *RTEL1* genotypes in the case and control subjects. Odds ratios (OR) and 95% confidence intervals (CI) were used to evaluate relative risk. All *p* values were two-tailed and *p* < 0.05 was considered statistically significant.
